# Innovations in Parkinson’s Disease: Strategies and Novel Technologies

**DOI:** 10.7759/cureus.87309

**Published:** 2025-07-05

**Authors:** Rehan Sha, Matheus A Dabus, Diego R Torres-Russotto, Nila Bhakuni, Guilherme C Dabus

**Affiliations:** 1 Neurosciences, Miami Dade College, Miami, USA; 2 Neurosciences, Baptist Health South Florida, Miami, USA; 3 Neurology, Baptist Health South Florida, Miami, USA; 4 Innovation, Baptist Health South Florida, Miami, USA; 5 Interventional Radiology, Baptist Health South Florida, Miami, USA

**Keywords:** artificial intelligence in medicine, assistive technology, functional brain imaging, parkinson’s disease, physical therapy rehabilitation

## Abstract

Parkinson’s disease (PD) is the second most common neurodegenerative disease, with its incidence expected to increase significantly in the next few years. Due to its unique pathophysiology and clinical features, the path to early diagnosis and treatment is challenging. Therefore, finding solutions that can address the disease process from every angle, whether improving patients’ quality of life or developing new methods for earlier diagnosis and treatment, is important. In this article, we review the pathophysiology of PD and examine the current and new technologies, how they are currently being applied, and the potential impact of their development on the future of Parkinson’s research and treatment.

## Introduction and background

Parkinson’s disease (PD) is a chronic, progressive, long-term, heterogeneous disorder with no cure. The number of affected individuals has been increasing throughout the last 30 years, currently affecting over 9 million people worldwide, resulting in over 200,000 deaths [1-3]. In the United States, approximately 900,000 to 1,000,000 individuals are affected by PD [1,4]. This number is projected to increase by 20% over the next five years [2]. PD is more prevalent in men with a male-to-female ratio of approximately 1.5 [1,3,5]. PD is uncommon before the age of 50 years, usually developing around 60 years of age, peaking in prevalence between 80 and 90 years of age [1,3,5]. The increase in PD has been associated with aging populations, better diagnosis and awareness, and increased environmental exposures [5].

PD is difficult to manage for several reasons. First, the Parkinsonian deterioration and potential for alpha-synuclein accumulation can exist for years or even decades before a patient demonstrates significant symptoms of the disease [6]. Additionally, a few traits differentiate PD from other diseases with similar symptoms and progression [7]. The lack of conclusive testing only makes the administration of earlier, effective treatment more difficult [8]. Further, with no disease-modifying therapies available, the neurodegenerative process continues inexorably.

Addressing PD is a matter of public health. PD symptoms affect the ability to walk, speak, or think properly, limiting the patient’s ability to perform routine daily tasks and activities, as well as limiting their social interactions, resulting in a major societal and economic burden. Approximately 52 billion dollars of the US budget annually is used in PD healthcare costs [2]. The cost per patient of medical treatment averages $2,500 a year, with surgery costing up to $100,000 per person [5].

Given the expected rise of PD as the life expectancy of the population increases, it is imperative to find solutions that can address the issue from every angle. Through an in-depth analysis of current research being done, initiatives being pursued, and startups across the world being funded, we have collected and evaluated some possible new technologies that could positively impact PD’s management. These solutions are designed to address the array of consequences of PD progression, alleviating both the physical and economic burden on individuals and populations worldwide.

## Review

Methodology

A wide literature search including terms encompassing innovations in the diagnosis, management, and treatment of PD was performed using PubMed. The research included original reports and comprehensive literature reviews. All abstracts were subsequently reviewed, and full articles were selected for final review when appropriate. The selected manuscripts were then included as references in the manuscript if they demonstrated innovative management options (diagnostics or treatment) for PD. If there was disagreement among the authors regarding manuscript inclusion, consensus was reached through discussion based on the relevance of data and scientific rigor. Some of the information about the startup companies mentioned in this manuscript was obtained from publicly available data. Lastly, additional information used mostly for general statistics was obtained from publicly available information from private organizations that perform independent research, such as the Parkinson’s Foundation.

Pathophysiology

PD occurs as a result of widespread depletion of dopamine and dopaminergic systems in the brain in conjunction with neural inflammation [6,9]. This is particularly emphasized in the substantia nigra pars compacta, a region of the basal ganglia that regulates motion and the reward system. As a primary gateway to some of the brain’s most complex functions, its deterioration can have catastrophic effects on brain function [8,9].

The primary process in PD is the selective loss of dopamine neurons and accumulation of Lewy bodies and alpha-synuclein aggregates [6,8]. The aggregates then become misfolded and inhibit neurons’ capacities to send and receive signals to the point of cell death. This alpha-synuclein congregation has also been found to interfere with the electron transport chain and general mitochondrial function, which is another significant indicator of PD. In some cases of mutation, the mitochondria can increase the production of reactive oxygen species (ROS) and free radicals, causing oxidative stress (OS), leading to excessive cell death, reduced ATP synthesis, and inflammation [10,11]. Additionally, there are several genes involved in PD. These include *SNCA*, which is linked to both familial and sporadic PD and is connected to the aggregation of alpha-synuclein. Mutations of the *LRRK2* gene are the most common genetic cause of autosomal dominant PD and are believed to inhibit autophagy, the process by which cells break down nutrients, recycle cellular components, and dispose of unusable waste. The genes *PINK1* and *PARK2* are connected to mitochondrial and ROS homeostasis, and their deterioration is strongly correlated with PD-related OS [11].

Early PD usually affects the medulla and the olfactory bulb, resulting in rapid eye movement sleep disorder and loss of or decreased smell [8,10]. These early changes often precede the onset of motor symptoms by several years. With the progression of the disease, the substantia nigra pars compacta, midbrain, amygdala, and temporal limbic cortex are affected and result in the usual PD motor symptoms as well as cognitive impairment and hallucinations [8-10].

The primary method for identifying PD is through the motor symptoms of Parkinsonism. These include impaired gait, bradykinesia, rigidity, and resting tremors. Bradykinesia presents as akinesia, hypokinesia, and slowness, and it is a required criterion. These symptoms are further expressed through gait abnormality, speech impairment, loss of arm swing, micrographia, and abnormal truncal posture. In addition, patients can experience cognitive impairment, sleep disorders, and gastrointestinal abnormalities. It must be noted, however, that the diagnosis is usually made when the disease has already advanced. The prodromal phase can last up to 12-14 years, and by the time the diagnosis is confirmed, significant neurological impairment will have already occurred [12].

The degeneration of the cortical cholinergic network also seems to be implicated in the pathophysiology of PD particularly through loss of cholinergic neurons and the involvement of the nucleus basalis of Meynert, which leads to a decrease in the levels of acetylcholine in the cortex, as well as the involvement of the pedunculopontine nucleus, which regulates cholinergic input to the brainstem and thalamus [13,14]. The involvement of the cholinergic system may occur early in PD and may affect the automaticity of gait, posture, and cognition [13].

Finally, there are a multitude of toxic substances that can increase the risk of PD. Chemicals such as 1-methyl-4-phenyl-1,2,3,6-tetrahydropyridine, rotenone, and paraquat, when in the body, can result in dopaminergic cell damage and impairment of mitochondrial function, leading to Parkinsonism [9].

Pharmaceuticals

The use of levodopa has been a cornerstone in the treatment of PD. The Parkinson Study Group examined the effect of levodopa against that of a placebo and found that those on daily dosages of levodopa had a significantly lower score on the Unified Parkinson Disease Rating Scale (UPDRS) at 42 weeks [15].

During the earlier stages of the disease, nearly all symptoms are easily manageable through regularly administered medications such as levodopa. However, as the disease progresses, its effectiveness varies over time, a phenomenon called motor fluctuations. With these fluctuations, there are variations in symptom control resulting in periods where the symptoms are well-controlled (ON state) and not well-controlled (OFF state). Furthermore, during the advanced stages of the disease, medications tend to cause more adverse effects, including dyskinesias [16]. In an attempt to reduce motor fluctuations, several pharmaceutical innovations have been developed, allowing levodopa and other medications to have a longer duration of benefit through slow absorption or decreased drug elimination.

One of the biggest challenges in PD care has been the complexity of absorption and the factors that alter the bioavailability of the medications. The most notable of these challenges is the blood-brain barrier (BBB), a highly selective membrane that surrounds the central nervous system. It is meant to protect the brain from foreign molecules, a function intended to prevent the invasion of pathogens. However, the BBB has proven to be a formidable obstacle to the passage of medication [17]. In addition, *Helicobacter pylori* infection has long had an association not only with the incidence of PD but the prevention of levodopa absorption. A study by Nyholm and Hellström demonstrated that eradication of *H. pylori* decreased the onset time and increased the duration of the levodopa effect when compared to pretreatment measurements, demonstrating that *H. pylori* may reversibly affect levodopa absorption [18]. Another factor that impacts the effectiveness of medications is gastroparesis, the impairment of motility in the gastrointestinal system, which prevents the stomach from emptying its contents properly. Gastroparesis results in the reduction of levodopa absorption, limiting its control of motor symptoms [19,20]. Once levodopa is absorbed, it would be rapidly metabolized by aromatic L-amino-acid decarboxylase (DOPA decarboxylase or DDC), an enzyme that leads to the biosynthesis of dopamine from L-DOPA. This would cause significant peripheral dopamine, which not only cannot trespass the BBB but would also cause significant side effects such as nausea. Carbidopa inhibits DDC, which then allows for more levodopa availability and less nausea.

Recent technological advancements that address these barriers seem to have focused on using nanotechnology to navigate the brain and introduce drugs more efficiently. In a study by Hawthorne et al. on the application of nanomedicine in PD, nano-manipulation (the movement or alteration of objects at the molecular scale) was able to significantly improve treatment outcomes [21]. The benefits of using nanoparticles to treat the disease included longer medication half-life, reduction of negative side effects, and stable drug concentrations [21]. Another study reviewing the management of PD corroborated the positive effects of nano-treatments such as polymeric nanoparticles that, when added to certain medications, can modify and cross the BBB [22]. Another promising form of treatment with nanotechnology is lipid nanoparticles, which allow a less toxic therapeutic approach due to their specific properties, such as bioacceptability, biodegradability, and lipophilic nature. They are able to cross the BBB in a less invasive way than other treatments, “exploiting” the natural entry provisions that the barrier establishes [23].

The use of genetic therapies in dopaminergic cell reproduction has also been explored. Recent studies have shown that by repurposing the stem cells’ property of unlimited replication, it is possible to produce new dopamine, which can help curb motor symptoms. Moreover, gene-altered stem cells are less foreign to the body than medications such as levodopa, which increases the chance of BBB crossing [24]. While several of the solutions here focus on alternatives to levodopa, there is also merit in finding new ways to administer the medication. While not currently approved by the FDA, ND0612 has gone through extensive trials and proven the efficacy of subcutaneous infusion of levodopa. In a study by Espay et al., use of ND0612 resulted in significant improvements for patients, including reduced off time and relatively better MDS-UPDRS (Part II) scores. While a variety of adverse effects necessitate more improvement on this treatment modality, it demonstrates the effectiveness of subcutaneous infusion, which can bypass gastric involvement and thereby avoid the complications caused by amino acid competition and bacterial overgrowth [25].

Another alternative to orally administered levodopa is intestinal gels. A study by Murata et al. reported the ability of levodopa-carbidopa intestinal gel (LCIG) to provide consistent concentrations of levodopa directly into the jejunum, which, in turn, reduces the risk and severity of dyskinesia. Among Asian subjects studied, off time was reduced by 4.6 hours after 12 weeks of LCIG treatment, in addition to notable reductions in motor fluctuations [26]. The US pivotal trial of LCIG was a 12-week, double-blind, randomized clinical trial that compared DUOPA to carbidopa-levodopa immediate-release tablets, demonstrating a significant reduction in off time without dyskinesias [27].

In 2024, the FDA approved the first subcutaneously administered levodopa formulation using foscarbidopa/foslevodopa. The pivotal studies demonstrated superior improvement in on time without troublesome dyskinesia, compared to oral immediate-release carbidopa/levodopa [28,29]. This has allowed patients to receive 24-hour infusion and reduce motor fluctuations, off times, with improvement of multiple outcomes, including sleep. This prodrug is enzymatically converted to levodopa and carbidopa in the body, reaching steady levels two hours after administration. A single-arm, open-label, phase 3 study with 244 patients demonstrated promising results with improvement in motor symptoms, sleep, and quality of life over one year with a reasonable safety profile (adverse events were mostly related to the infusion site) [25].

Yet another option with promising results is apomorphine continuous subcutaneous infusion. Apomorphine is a dopamine receptor agonist derived from acidification of morphine, with similar effects on Parkinson’s symptoms compared to levodopa. The TOLEDO trial was a multicenter, randomized, double-blind, placebo-controlled study that randomized 107 PD patients with persistent motor fluctuations. The trial results demonstrated that patients treated with apomorphine subcutaneous infusion showed a statistically significant greater reduction in off time with no increase in dyskinesia and a good safety profile [30].

There are many more initiatives and startups that are focusing on other compelling methods and products, as shown in Table 1.

**Table 1:** Startups and initiatives developing pharmaceutical-based solutions.

Company	Product/Method	Description
Neuroderm	ND0612	Currently developing product ND0612 for Parkinson’s disease patients with motor symptoms. The device would regularly self-administer doses of levodopa and carbidopa [31]
Synnerva	Central nervous system drug composition	Making drug compositions to affect the central nervous system, allowing for a significant reduction of the necessary dose and elimination of side effects [32]
Cytora	Oral mucosa stem cell therapy	Trying to standardize the use of off-the-shelf stem cell therapies and medicines, derived from adult human oral mucosa stem cells [33]
Brainstorm Cell Therapeutics	Autologous stem cell therapeutics for neurodegenerative disorders	Treats patients with amyotrophic lateral sclerosis and multiple sclerosis with their product, NurOwn, which “transforms autologous mesenchymal stem cells into neurotrophic factor-secreting cells” [34]

With any new pharmaceutical development, it must be kept in mind that further testing and research are necessary before these become viable or even medically standard treatments for PD. All of the discussed advancements in this section are testament to the progress of medical science research, but are worth more exploration due to factors such as FDA approval, negative side effects, and non-representative study populations. Regardless, they represent a better outlook for PD as they continue to be developed.

Physical therapy

Enhancing physical activity is critical in the treatment of PD. Multiple studies have shown that forms of exercise and daily physical activity can improve the symptoms of the illness. When it comes to the physical degradation caused by PD, bradykinesia and freezing of gait (FOG) are important causes of concern [35]. The American Parkinson Disease Association lists falling as the number one symptom that increases the risk of death, citing it as “one of the major causes of emergency room visits and hospitalizations for people with PD.” While levodopa can improve these motor symptoms in the early stages of the disease, the incidences of FOG and falls increase as PD progresses [36]. Therefore, finding alternative treatment methods through physical therapy and technology is necessary to create sustained gait improvements in individuals with PD.

Assisted technology (AT), as defined by Capato et al. in their breakdown of gait-related technology, includes the systems and services related to the delivery of assistive products (APs) used to promote the inclusion and participation of persons with disabilities. It is possibly one of the most effective methods by which Parkinson’s patients can regain independence and quality of life. The goal of any given AP can range from diagnosis to providing physical therapy plans from collected data on a user’s body [37].

One promising form of AT is exoskeletons. In a comprehensive exercise and physical performance study, Gryfe et al. found that Parkinson’s patients who used an exoskeleton over an eight-week period performed better in certain physical benchmarks used to assess the progression of the disease than those without. Among these benchmarks are the Scales for Outcomes in Parkinson’s disease-Cognition (SCOPA-COG), a cognitive function test, and the six-minute walking test (6MWT), which showed their physical performance and any gait impairments [38]. Startup companies are focusing on a variety of personal, adaptive devices, including a glove that can improve hand muscle control and a treadmill-like system that trains balance and steady walking. While a lot more work needs to be done to ensure user safety and overall efficacy, these technologies have a lot of potential for reducing tremors and gait complications in Parkinson’s patients. Similar approaches are being taken by groups such as ReWalk, NexStride, and the Boston University Center for Neurorehabilitation.

Another relatively recent solution for Parkinson’s is the use of virtual and augmented reality (VR and AR), which uses simulated three-dimensional spaces to run physical therapy routines with little to no equipment besides a headset. In a review of how VR is being applied to rehabilitation, Kwon et al. analyzed randomized controlled trials consisting of over 500 participants who underwent VR training. Their performance was measured against a control group using standards such as the 10-meter walk test (10MWT), the 6MWT, the MDS-UPDRS, and the Freezing of Gait Questionnaire (FOGQ). Overall, it was found that VR was an effective tool for rehabilitation that showed substantial improvement in balance and gait. The authors specifically noted that “incorporating game-like elements and other interactive features can increase patient enjoyment and willingness to participate in training sessions,” as it gives patients more motivation and unique, customized approaches to their treatment [39]. If developers can figure out how to remove safety risks such as real-life collisions and enhance spatial awareness, particularly for VR, this treatment modality could very well make it into standard or daily use for many patients.

Other groups that are developing physical therapy technology to be applied to Parkinson’s include Conflu3nce, MediTouch, NeuroDerm, Neurogait, and Neurosteer, as seen in Table 2.

**Table 2:** Startups and initiatives developing physical therapy-based solutions.

Company	Product/Method	Description
Conflu3nce	Personalized solution for detecting cognitive health changes	Developing a system that is meant to address cognitive or motor issues for people of all demographics, assisting them with any mental/physical disorders they are having. The system can be used for early detection by capturing cognitive expression and begin working to remedy the symptoms [40]
MediTouch	Physical therapy device	Manufactures a suite of devices focused on assisting individuals with self-guided physical therapy [41]
NeuroDerm	Drug-device combination solutions for central nervous system disorders	Currently developing product ND0612 for Parkinson’s disease patients with motor symptoms. The device would regularly self-administer doses of levodopa and carbidopa [42]
Neurogait	Monitoring Parkinson’s using artificial intelligence	Developing a clinical monitoring platform that tracks movement and biological markers to provide a base for clinicians to make treatment plans for Parkinson’s patients [43]
Neurosteer	Remote brain activity monitoring and assessment platform	Creating a platform consisting of a wearable device and cloud system that will provide real-time assessment of the brain, focusing on finding biomarkers for mental disorders such as dementia and Parkinson’s [44]
Lifeward	Rewalk personal exoskeleton technology	Provides services to patients with spinal cord injuries that allow them to function physically and independently. Their Rewalk Exoskeleton is cited by Lifeward as the most widely used and first FDA-approved exoskeleton technology currently on the market [45]
NexStride	NexStride walking assistance suite of devices	Prevents people with neuromuscular or neurodegenerative conditions from losing mindfulness or consciousness during movement by playing audio and visual cues from their walking device [46]

Artificial intelligence

Artificial Intelligence (AI) represents a burgeoning technology with immense potential. In the context of PD, early detection poses a significant challenge for medical providers. Detecting PD in its initial stages is crucial as it allows for timely intervention, effectively mitigating its debilitating effects. However, the complexity of PD makes early diagnosis elusive, complicating treatment decisions.

AI can potentially assist with a more personalized approach regarding diagnostic and management strategies. Furthermore, advancements in AI have streamlined these diagnostic and treatment challenges, transforming what were once daunting obstacles for non-specialists into more routine clinical procedures [46].

New AI technologies have been specifically developed to diagnose PD early. In a study by Islam et al. on the application of AI algorithms to detect the severity of PD, the authors concluded that, due to the algorithm’s mean absolute error in detecting the severity of PD being statistically far superior than that of two certified raters (0.58 to 0.83) and being similar to that of three expert neurologists (0.58 to 0.53), AI algorithms can accurately assess the severity of PD. The algorithm in this study was able to grade the severity of PD on a scale of 0-4 following the MDS-UPDRS [47]. The benefits of using AI for diagnosis are the accessibility of having near-expert-level detection at any site, allowing for diagnostic efficiency. This could facilitate screening and subsequent referrals to the neurologist.

Some promising new technologies use oculometric markers to distinguish early PD from other Parkinsonians through eye movements, pupil responses, and blink rate. Some of the diagnostic technologies have the potential to guide effective treatments tailored to the patient through more accurate monitoring of disease progression. To validate this idea, the Parkinson’s Longitudinal Oculometric Measurement Assessment (PALOMA, NCT05862649) has been launched. PALOMA is a multicenter longitudinal study of 300 patients with idiopathic PD that aims to evaluate the correlation between oculometric measures and clinical assessment over time, as well as the potential to detect early change in clinical status using an oculometric assessment [48].

Several companies are developing products that make use of AI, aiming to not only improve the detection of PD but to improve life overall for patients with PD (Table 3).

**Table 3:** Startups and initiatives developing artificial intelligence-based solutions.

Company	Product/Method	Description
ActualSignal	Parkinson’s care management platform	Leverages artificial intelligence to more efficiently and comprehensively create personal disease profiles and enhance patient care for those with Parkinson’s [49]
AnyCan Technology	Mobility and balance platform	Developing a platform, STEADY, that analyzes a patient’s gait and posture to help them correct it, acting as a form of physical therapy [50]
Panacea-ML	Machine learning tool for streamlining clinical trials	Gives pharmaceutical companies insights into current drugs and treatments so that more effective treatments can be brought to the market faster [51]
Qrons	Advanced stem cell-based solutions for neuronal injuries	Currently developing products for traumatic brain injuries using stem cells and hydrogels [52]
Umoove	Face and eye tracking technology	Facial and eye-tracking software for mobile devices, with an interpretation layer that turns the movements into an interactive language capable of providing valuable data. This information can give insights into brain activity [53]

While these technologies could revolutionize the way PD is diagnosed, it is important to recognize potential setbacks and limitations. AI and machine learning are subject to issues with privacy, security, and training bias. Events from the past decade have revealed that the technology is still being perfected, and is not necessarily safe for handling secure medical information. If one is considering utilizing any AI-based method discussed in this review, it is critical to understand what information a service requires, how much of that data gets reused for training their model, and what exactly it is used for. With many of these modalities being startups and in the early phases of their development, cybersecurity risks are sure to be present.

Software as a service

Innovators have been starting to look to software as solution for modern problems. Known as software as a service (SaaS), these new programs are designed to enhance quality of life. In the context of PD, different software can be developed to help with several problems associated with PD, such as dyskinesia and slurred speech [54]. Furthermore, software can also be used to monitor patients’ brain health through a mobile app. The accessibility and convenience of software promises to be a long-lasting opportunity in medicine.

In a study conducted by Palacios-Alonso et al. on the application of a software to oversee and examine patients with neurodegenerative diseases remotely, the authors concluded that software can be used for “Parkinson’s clinical monitoring and patient management using speech, movement, and acoustic stimulation.” The authors came to this conclusion by sending raw speech recordings to the application, Monitoring Parkinson Using Locution (MonParLoc). The recordings were then processed by BioMet® Phon, an application that supplies feature extraction capabilities. This methodology was tested with 45 volunteers, and it was shown that acoustic recordings can be used to create features, traits, and useful ratios in remotely observing PD patients [55].

Several companies are releasing applications available to anyone with a mobile device to use the sensors already present in smartphones. One example is Mon4t, which performs complete data analysis using cloud-based solutions, extracting medical biomarkers using proprietary algorithms, and delivering AI-based insights. It is encouraging that some of these products are FDA approved, further confirming the authenticity of the application [56]. Another company, Razmobility, and its software, Voiceitt, uses speech recognition technology to turn non-standard speech patterns into clearer speech in real time, allowing people with severe speech impairments to communicate with others. To use the software, the user must train the application to understand their voice by repeating phrases several times. Once training is done, the user can speak to their phone with their own voice, and the application will translate and show the user’s speech on the screen [57]. The benefits of these types of applications are that they allow people with slurred speech to communicate with anyone. Additionally, physicians can monitor their patients’ health through a mobile device (Table 4).

**Table 4:** Startups and initiatives developing software-based solutions.

Company	Product/Method	Description
Mon4t Clinic	Brain monitor and remote treatment app	Developing brain monitors for patients and physicians to actively see their brain health [58]
Voiceitt	Voice recognition technology for non-standard speech	Uses speech recognition technology to turn non-standard speech patterns into clearer speech in real time, allowing people with severe speech impairments to communicate with others [59]

A vast majority of these proposed solutions cannot clinically treat the disease, but they offer a new way to help patients keep track of their condition and improve quality of life using modern developments in software and mobile hardware. Of course, as mentioned before with AI, there is still a large margin of error for the accuracy and precision of the data that a phone or mobile device can collect. Yet, as mobile technology becomes more sophisticated, we hope that this error can be significantly reduced in the future.

Surgical and other advanced therapies

As PD symptoms advance, increasing medication dosage might be needed to address the symptoms. However, sometimes this is not possible due to side effects [2,3]. This state is known as medical failure. To reduce off periods and dyskinesia, surgical and advanced therapies may be used. Deep brain stimulation (DBS) has been used for over 30 years in the treatment of primarily motor fluctuations in advanced PD. This treatment involves the implantation of leads in the brain that electrically stimulate specific target regions, including the subthalamic nucleus and globus pallidus interna [2,4,12]. The main indication for the surgery is medical failure. However, ideal candidates are considered those younger than 70 years , patients with on-off fluctuations, adequate dopaminergic response, dyskinesia, and with good cognitive function [60]. Another important indication is medication-resistant tremor. Weaver et al. compared DBS versus best medical treatment in patients with advanced PD. Its primary outcome included time spent in the on state without troubling dyskinesia. The study demonstrated that patients treated in the DBS arm gained more on time without troubling dyskinesia compared to the best medical therapy arm (p = 0.001). Patients treated with DBS also showed improved motor function and quality of life despite higher serious adverse events compared to best medical therapy [61]. A randomized study comparing bilateral pallidal stimulation with subthalamic stimulation in 299 patients demonstrated similar improvement in motor function. The occurrence of serious adverse events was similar in the two groups. However, the level of depression favored pallidal stimulation compared to subthalamic stimulation [62]. Another study examining the 36-month outcomes after DBS for PD using motor function, quality of life, and neurocognitive function as outcomes demonstrated improved motor function as well as quality of life, although improvement decreased over time. There was a decline in overall neurocognitive function, likely from disease progression; however, the decline was faster for patients with subthalamic stimulation compared to pallidal stimulation [63].

The response to DBS may vary depending on the patient population. Some patients may not improve significantly with DBS, particularly older patients, patients with dementia and other cognitive impairments, severe autonomic dysfunction, and patients with medication-resistant symptoms. Patients with tremor-predominant Parkinsonism who are not bothered by bradykinesia or rigidity may benefit from thalamic DBS or high-intensity focused ultrasound (HIFU) lesional thalamotomy [64]. Although uncommon, due to its invasive nature, DBS risks include infection, hemorrhage, neurological deficits, and, eventually, death [65].

A particularly effective recent technology is focused ultrasound (FU). As mentioned above, this procedure is particularly helpful in the management of medication-refractory, tremor-dominant PD. In this procedure, the patient’s scalp is shaved, and a headframe is placed. The patient is then positioned in the MRI scanner, and therapeutic FU waves are administered with increasing energy, targeting a specific part of the brain (thalamus). These sound waves raise the temperature of the targeted cells to such an extent that the tissue is ablated. The target can be adjusted based on clinical feedback. Bond et al. demonstrated that FU improved tremors compared to the sham procedure. What makes this technique particularly advantageous is that it is considered less invasive than DBS. Nonetheless, the procedure is usually performed unilaterally due to possible adverse events, which are not rare, although mostly non-disabling [66].

Both HIFU and DBS have been used in advanced PD and have similar risks for complications. The limitations of HIFU include the unknown long-term outcome, relatively new nature of the treatment, the irreversible nature of the side effects and injury, the need to shave the head completely (which some patients refuse to do), and the fact that not all skull types allow for the therapy to be used. Although DBS has proven benefit for many years in PD care, further limitations include the required need for more visits (as the system needs to be programmed), some changes in lifestyle when carrying a device in the body, the battery needs to be changes over time, among others. Therefore, these therapies are individualized to every patient.

Another surgical option for advanced PD patients unresponsive to oral treatment consists of continuous pump infusion of medication such as LCIG. The gel is administered continuously and directly to the patient’s jejunum using an infusion pump via percutaneous jejunostomy [67]. Othman et al. studying the pharmacokinetics of levodopa demonstrated that this technique allows for faster absorption, reduced variability, and more continuous plasma levels compared to oral medication [68]. Its clinical effectiveness has also been demonstrated. A multinational, observational, cohort study demonstrated improvement in off periods as well as dyskinesia duration and severity with improvement in quality of life [68]. Another study demonstrated that LCIG improved motor symptoms, and most patients reported improvement in their quality of life, autonomy, and clinical global status [69]. It is important to note that in one study, approximately 40% of patients developed significant deterioration of cognitive function [70]. An alternative to LCIG is levodopa/entacapone/carbidopa intestinal gel, which has been shown to have similar pharmacokinetics with higher levodopa levels. As discussed previously, the recent introduction of subcutaneous foscarbidopa/foslevodopa may change the treatment algorithm of patients with motor fluctuations.

Future use of current technology

MRI has been used to diagnose neurological diseases for the last four decades. However, its use in the diagnosis of PD has been limited. Currently being explored is the use of MRI with a variety of different scopes to find the correlation between certain chemicals or structures and the incidence of PD [71]. For example, studies have shown that an abnormal increase of iron in substantia nigra can set Parkinson’s patients apart from non-afflicted individuals, and therefore, iron-sensitive neuroimaging could possibly result in earlier diagnoses. Studies have demonstrated that iron could even be present in the prodromal phase, allowing PD diagnosis and treatment before severe and debilitating symptoms arise [72]. If this scope were shifted toward neuromelanin, however, further information could be acquired from dopaminergic cells such as L-DOPA. By assessing neuromelanin concentrations with MRI, neuroscientists have been able to determine the severity and timeline of an individual’s disease [73]. Additionally, studies in diffusion tensor imaging (DTI) demonstrate promise in examining the brainstem for metrics strongly related to Parkinson’s. Among these metrics are mean diffusivity and white matter loss, which are effectively examined through DTI and are indicators of disease duration and severity, respectively [9]. It is important to note that most of the above-mentioned techniques are simply trends found in research and imaging studies, so while they are not in standard medical practice at the moment, it is entirely possible that their status will change in the near future.

## Conclusions

PD is a debilitating disorder that has been a significant personal and societal burden for several decades. Advancements in the diagnosis and treatment of the disease and its major biological factors have been slow. Recent innovations utilizing breakthrough strategies such as AI, digital methods to increase accessibility and efficiency, technologies that improve drug delivery, technologies that enhance the effect of physical therapy, and new minimally invasive procedures have shown promising results. The strategies described here have the capability of enhancing clinicians’ ability to understand the disease better, make earlier diagnoses, start treatments sooner, and, most importantly, improve the quality of life of PD patients. As future advancements bring technology closer to the healthcare sphere, the reduction of PD burden looks increasingly promising.
